# Perioperative Blindness in Spine Surgery: A Scoping Literature Review

**DOI:** 10.3390/jcm13041051

**Published:** 2024-02-12

**Authors:** Jacob Sperber, Edwin Owolo, Tanner J. Zachem, Brandon Bishop, Eli Johnson, Eleonora M. Lad, C. Rory Goodwin

**Affiliations:** 1 Department of Neurosurgery, Duke University School of Medicine, Durham, NC 27710, USAeli.johnson@duke.edu (E.J.); 2 Department of Ophthalmology, Duke University School of Medicine, Durham, NC 27710, USA; 3 Department of Mechanical Engineering and Materials Science, Duke University, Durham, NC 27710, USA; 4 College of Medicine, Kansas City University; Kansas City, MO 64106, USA; 5 Duke Cancer Institute, Duke University Medical Center, Durham, NC 27710, USA

**Keywords:** perioperative vision loss, spine surgery, complication, risk stratification, ischemic optic neuropathy, central retinal artery occlusion, cortical blindness

## Abstract

Perioperative vision loss (POVL) is a devastating surgical complication that impacts both the recovery from surgery and quality of life, most commonly occurring after spine surgery. With rates of spine surgery dramatically increasing, the prevalence of POVL will increase proportionately. This scoping review aims to aggregate the literature pertinent to POVL in spine surgery and consolidate recommendations and preventative measures to reduce the risk of POVL. There are several causes of POVL, and the main contribution following spine surgery is ischemic optic neuropathy (ION). Vision loss often manifests immediately following surgery and is irreversible and severe. Diffusion weighted imaging has recently surfaced as a diagnostic tool to identify ION. There are no effective treatments; therefore, risk stratification for counseling and prevention are vital. Patients undergoing prone surgery of long duration and/or with significant expected blood loss are at greatest risk. Future research is necessary to develop effective treatments.

## 1. Introduction

Perioperative vision loss (POVL) is a rare yet incredibly disabling phenomenon, often leading to irreversible vision loss in an already vulnerable patient population. POVL commonly occurs after spine surgery, with rates ranging from 0.03% to 0.2% [1,2,3,4]. Visual acuity has been strongly correlated with physical activity levels, which has profound implications for the recovery process following spine surgery [5,6,7]. Dramatically increasing rates of spine surgery in the last 10 years suggest that the prevalence of POVL will increase proportionately, thus warranting research into this complication [8,9,10].

In the spine surgery patient population, POVL was originally reported in 1948 [11]. Several case reports in the early 1950s verified this finding of vision loss following surgery, hypothesizing pathophysiological contributions from systemic hypotension, malpositioning, and anesthetics [12,13,14]. More recent investigations describing POVL saw a shift from qualitative case reports to quantitative epidemiological studies. In 2009, Shen and colleagues analyzed 5.6 million patients from the Nationwide Inpatient Sample and found an increased incidence of POVL in patients undergoing spine and cardiac surgery at 0.09% and 0.03%, respectively [2]. These findings are corroborated by a population-based study from 2008 noting an incidence of visual disturbance following spine surgery of 0.094% and a retrospective review from 1997 describing loss of visual acuity in 0.20% of patients undergoing spine surgery [3,4]. In particular, patients undergoing scoliosis correction or posterior lumbar fusions were noted to have the highest rates of perioperative vision impairment [3]. A recent comprehensive report of complications following adult deformity surgery found that three-column osteotomy procedures (3CO) have double the rate of visual acuity changes compared to non-3CO procedures, 0.4% and 0.2%, respectively [15].

Several studies have documented the existence of perioperative blindness following spine surgery; however, few reviews exist consolidating the known literature. Furthermore, the pathophysiological mechanisms underlying POVL have been studied in more detail in recent years; thus, a review of recent literature is warranted. This review will summarize existing case reports as well as aggregate conclusions from previous reviews to present a modern perspective on POVL as well as discuss preventative measures that may be taken to reduce the risk of vision loss in vulnerable populations undergoing spine surgery.

## 2. Materials and Methods

We searched the PubMed database (Bethesda, MD, USA) for English-language studies relevant to POVL and spine surgery. A scoping review was conducted in accordance with the Preferred Reporting Items for Systematic Reviews and Meta-Analyses Extension for Scoping Reviews (PRISMA-ScR) checklist [16]. A scoping review was selected instead of a systematic review due to the strengths of scoping reviews in both identifying knowledge gaps in the field and clarifying information [17]. The PubMed search strategy employed is described in the Appendix A and yielded 310 articles. Selected articles were included in this manuscript if blindness or vision loss manifested during or after spine surgery. Title and abstract screens excluded articles that did not address POVL in the setting of spine surgery. Non-English studies and non-human studies were excluded from the analysis. The references of the previously published reviews selected were also manually searched. A total of 78 articles were included in this scoping review. The most recent search was conducted on 8 December 2023.

## 3. Discussion

### 3.1. Historical Narrative of Blindness in the Literature

The first cases of POVL after spine surgery were documented in the late 1940s and significant progress in understanding both the pathology and risk factors has been made in the last 80 years. The main historical findings across various time periods are summarized in Table 1.

#### 3.1.1. 1940–1990

Slocum, O’Neal, and Allen reported the first case of blindness after spine surgery in 1948 when a patient developed blindness after improper positioning using a Bailey headrest [11]. Givner and Jaffe reported an individual who developed blindness due to central retinal artery occlusion (CRAO) several hours following choledochojejunostomy for pancreatic carcinoma [12]. Gillan reported two patients in 1953 who developed unilateral blindness upon awakening from anesthesia, one from CRAO and the other of unknown origin, neither of whom recovered their vision [13]. The patient who developed CRAO was operated on due to pyelonephritis caused by a calculus impacting the right ureter and the other patient had hepatocellular carcinoma [13]. Hollenhorst reported eight patients who developed blindness following neurosurgical procedures and corroborates these findings in a series of studies using monkeys to further interrogate the pathophysiological mechanism [14]. Little, in 1955, reported 27,930 cases of deliberate hypotension during anesthesia procedures and describes three cases of retinal ischemia [18]. Bradish and Flowers reported a 12-year-old girl who underwent a two-stage spinal fusion for rapidly progressing scoliosis associated with osteogenesis imperfecta [19]. The operation was performed under permissive hypotension and the patient awoke with a visual acuity of counting fingers (CF) in one eye, which deteriorated to light perception within two days, attributed to CRAO of the eye [19]. Aldrich and colleagues, in 1987, examined 15 patients with cortical blindness, and an additional 10 patients’ charts who previously presented with cortical blindness, to determine the causes of cortical blindness as well as the best techniques for diagnosing and managing the complication [20]. Shaw and colleagues sought to determine the etiology of postoperative neuro-ophthalmological complications using samples of individuals who underwent coronary artery bypass graft surgery and peripheral vascular surgery, consisting of 312 and 50 patients, respectively [21].

#### 3.1.2. 1991–2000

Grossman and Ward, in 1993, reported a child who developed CRAO after scoliosis surgery where a horseshoe headrest was utilized [22]. Katzman et al., in 1994, described a patient who experienced blindness following significant hemorrhage during lumbar spine surgery and suggested this was the first report of amaurosis attributable to an orthopedic operative procedure [23]. Shapira and colleagues, in 1996, aimed to determine why the incidence of anterior ischemic optic neuropathy (ION) increased from less than 0.5% to 1.3% following open-heart procedures at their institution and described several perioperative risk factors: prolonged bypass time, low hematocrit, excess weight gain, and the use of epinephrine and amrinone [24]. Sys and colleagues, in 1996, published a case report of CRAO in an adult after a lumbar spinal fusion [25]. In 1997, Stevens et al. conducted a retrospective review of 3450 patients undergoing spinal surgery and observed 7 patients that experienced postoperative loss of visual acuity [4]. Myers and colleagues, in 1997, identified 37 patients that developed visual acuity loss following spine surgery, with complex instrumented fusions complicated by hypotension and prolonged operating time constituting a breadth of the cases [26]. Dilger described a case of ION in a diabetic, obese patient that underwent a lumbar spine fusion [27]. Alexandrakis and Lam, in 1999, described a 68-year-old woman that experienced bilateral ION after prone spine surgery, which they surmised was attributable to pressure to the periorbital region [28].

#### 3.1.3. 2001–2005

Cheng et al. conducted an experiment recording intraocular pressure during various stages of prone spine surgery in 20 patients without eye disease and note that prone positioning is associated with an elevated intraocular pressure [29]. Lee and Lam, in 2001, reported a 58-year-old man undergoing posterior lumbar fusion without intraoperative complication who experienced unilateral vision loss diagnosed as posterior ION [30]. Roth and Barach published an Editorial View that accompanied Lee and Lam’s 2001 case report which summarized the then current knowledge pertaining to POVL and described additional work that would be necessary to better understand the complication [30,31]. Sadda et al., in 2001, conducted a retrospective review of patients with posterior ION and concluded that there are three main causes: perioperative, arteritic, and nonarteritic [32]. Dunker et al. compared 7 cases of posterior ION from their institution to 46 cases published in the literature, concluding that middle-aged males that underwent spine surgery with lengthy intraoperative hypotension, postoperative anemia, and facial swelling were at the greatest risk for developing posterior ION [33]. Deyo, Nachemson, and Mirza published in the New England Journal of Medicine a paper describing the increasing rates of spine-fusion surgery in the United States, and cited blindness as a complication of fusion surgery [8]. Halfon and colleagues, in 2004, presented two cases of complete ophthalmoplegia and CRAO following prone spine surgery [34]. Hayreh, in 2004, reports 42 patients with posterior ION, 3 of whom were classified as surgical-induced posterior ION, of which 1 underwent a posterior lumbar fusion [35]. Buono and Foroozan published a review in 2005 characterizing posterior ION, offering a comprehensive summary of the literature pertaining to spine surgery and posterior ION [36]. Ho et al. presented a review about POVL after spine surgery, with an emphasis on ION pathology [37]. Lee and colleagues described four patients treated in an intensive care unit that developed blindness secondary to ION (one patient who had both ION and traumatic optic neuropathy) and hypothesized that vasopressors may contribute to the loss of vision [38].

#### 3.1.4. 2006–2010

Lee and colleagues, in 2006, retrospectively analyzed 93 cases of POVL after spine surgery and found that ION was the most common cause in 83 of the patients [39]. Leibovitch et al. published, in 2006, a case report of an 80-year-old man who experienced unilateral blindness due to ischemic orbital compartment syndrome after a lumbar decompressive laminectomy [40]. The American Society of Anesthesiologists Task Force published a practice advisory on perioperative blindness in 2006 summarizing the literature and describing risk factors [41]. Mobley et al. presented a literature review of concerns related to prone positioning during surgery [42]. Walick and colleagues, in 2007, conducted an experiment to determine whether intraocular pressure increases in the prone flat versus prone Trendelenburg position and found that intraocular pressure is elevated when patients are positioned in the prone Trendelenburg position [43]. Roth, Tung, and Ksiazek described a case of CRAO in a 53-year-old man undergoing posterior lumbar fusion with the use of eye protectors [44]. Baig et al., in 2007, presented a literature review on POVL by outlining its pathogenesis as well as current gaps in the existing knowledge [45]. Patil et al., in 2008, conducted a national population-based retrospective cohort study of all patients from 1993 to 2002 that experienced ION, CRAO, or other postsurgical visual impairments following spine surgery [3]. Yu et al., in 2008, documented a case of blindness due to ischemic orbital compartment syndrome following prone spine surgery [46]. St-Arnaud and Paquin elucidated safe positioning practices for patients undergoing neurosurgical procedures and indicated that blindness may result if excess pressure is placed on the eyes [47]. Reddy et al., in 2008, reported a 55-year-old gentleman who awoke from a prone lumbar laminectomy with a visual acuity of CF in the right eye and hand motion in the left eye [48]. The patient was diagnosed with posterior ION and, 4 weeks later, visual acuity subsequently resolved to 20/25 in the right eye and 20/20 in the left eye with restricted peripheral visual fields [48]. Shen, Drum, and Roth, in 2009, outlined the prevalence of POVL in spinal, orthopedic, cardiac, and general surgery throughout a 10-year period, demonstrating an increased risk for POVL in both cardiac and posterior spine fusion surgeries [2]. Hayreh, in 2009, characterized the various types of ION, indicating that there are several reports of posterior ION during prolonged surgical procedures, such as spine cases [49].

#### 3.1.5. 2011–2019

Corda et al., in 2011, surveyed 437 patients (184 respondents) who underwent prone spine surgery and found that 80% of respondents would prefer full disclosure of POVL risk prior to the procedure [50]. The American Society of Anesthesiologists Task Force updated their 2006 practice advisory with detailed statements pertaining to preoperative patient evaluation and preparation, intraoperative management, the staging of surgical procedures, and postoperative management [41,51]. Goni et al., in 2012, described a 38-year-old male that underwent posterior lumbar decompression and instrumentation who awoke with bilateral blindness found to have bilateral occipital lobe infarcts and diagnosed with cortical blindness [52]. Ooi et al., in 2013, documented a 22-year-old male who underwent prone surgery for resection of a cervical extradural hematoma and awoke with central blurring of the right eye, which was diagnosed as CRAO [53]. Nickels, Manlapaz, and Farag, in 2014, discussed and expanded upon the findings from the American Society of Anesthesiologists Task Force’s practice advisory on POVL [54]. Sciubba et al., in 2015, aggregated 93 articles to present a thorough list of complications associated with adult spine deformity surgery, and 12 of 11,692 patients presented visual acuity changes [15]. Quddus et al., in 2015, discussed two cases of posterior ION that were not associated with spinal surgery, but acknowledged that prolonged surgical procedures, such as spine surgery, are common causes of posterior ION [55]. Kla and Lee, in 2016, offered an overview of POVL, indicating the spine surgery is one of the most common surgical causes of POVL [1]. Roth and Moss presented updated data describing perioperative ION epidemiology, presentation, and risk factors [56]. In 2019, the American Society of Anesthesiologists Task Force updated their 2012 practice advisory and included recommendations for management during the preoperative, intraoperative, and postoperative periods [41,51,57].

#### 3.1.6. 2020–Present

Wang, Brewer, and Sadun, in 2020, presented a review summarizing the literature on perioperative posterior ION and an experiment to ascertain which risk factors most strongly contribute to its development [58]. Chang, Chien, and Wu, in 2020, reviewed studies published prior to 2019 to determine which anesthetics influence intraocular pressure and concluded that propofol-based total intravenous anesthesia ameliorates increased intraocular pressure better than volatile anesthetics; two of the sixteen studies analyzed consisted of patients undergoing prone spine surgery [59]. Singh et al., in 2021, reviewed ophthalmic complications associated with perioperative anesthesia, citing literature indicating that spinal fusion surgeries are associated with both ION and CRAO [60]. Oliver et al., in 2021, reported a 9-year-old boy who was diagnosed with posterior ION following supine craniotomy for an epidural abscess [61]. Mulukutla, Yelemarthy, and Vadpalli, in 2021, presented a 46-year-old female who underwent anterior cervical discectomy and fusion and developed bilateral loss of vision 9 h after the surgery, and was found to have cortical blindness [62]. Ramakrishnan et al., in 2021, described a 26-year-old male who underwent prone fusion and instrumentation from C5 to T2 and immediately upon awakening from surgery presented unilateral, left-sided vision loss, which was diagnosed as CRAO [63]. In 2021, Kaur et al. performed an experiment to monitor ocular pressure changes during prone spine surgery and found that ocular pressure was significantly elevated at the end of the case when compared to baseline pressures [64]. Danyel et al. described the use of diffusion-weighted MRI to diagnose ION in patients with giant cell arteritis [65].

### 3.2. Pathophysiology

ION and CRAO are the most common mechanisms underlying POVL following spine surgery. Relative to ION, CRAO occurs less frequently; one retrospective analysis of 93 POVL cases after spine surgery found ION to be the primary cause in 89% of patients, whereas CRAO was the cause in 11% [18,39]. Cortical blindness has also been reported, although this is more common when spine surgery is associated with cardiothoracic surgery due to hemodynamic manipulations and subsequent increased risk for emboli [66,67,68].

#### 3.2.1. Ischemic Optic Neuropathy

ION is classified as anterior (AION) when affecting the optic disc and posterior (PION) when involving more proximal optic nerve and retrobulbar tissues [60]. Infarction of the small branches of the posterior ciliary arteries disrupting axoplasmic hemostasis is believed to underly AION [60,69,70,71]. PION describes intra-orbital infarction of the optic nerve, often stemming from hemodynamic complications or manual compression [60]. PION is the most common etiology of POVL in spine surgery [36]. Prone positioning has been shown to increase intraocular pressure (IOP) and may cause dependent pooling of fluid around the retina [29,42,46,47]. Subsequent large fluid boluses may increase orbital pressure and cause ischemic injury due to decreased perfusion to the optic nerve [37,40,43]. Orbital venous congestion similarly decreases arterial perfusion of the eye and has been observed in spine cases when patients have been in Trendelenburg [28]. Many studies have correlated anemia, hypotension, and hemodilution with ION, suggesting decreased ocular perfusion pressure inducing ischemic damage to the optic nerve [27,37]. The administration of vasopressors has also been correlated with ION, with one group reporting ION after lumbar fusion with constant phenylephrine infusion and other reports of associations concerning vasopressor infusion and ION [24,30,38].

#### 3.2.2. Central Retinal Artery Occlusion

The pathophysiology of CRAO relates to vascular occlusion attributable to retinal emboli, atherosclerosis, inflammation, or vasospasm [60]. Inadvertent pressure to the orbits due to prone positioning directly modulates intraocular pressure (IOP) and orbital arteriovenous pressure, causing arterial and episcleral venous congestion [11,14,19,48]. The relief of pressure induces ischemic vasodilation, which is accompanied by transudate leaking from the vasculature into the tissue and subsequent pathologic retinal edema [14,34]. CRAO has been documented following the use of both horseshoe and rectangular headrests, and paradoxically eye protectors due to incidental traumatic compression of the eye [22,25,44]. Additionally, as blood loss can be significant in certain complex spine procedures, the use of hemostatic agents such as transexamic acid and other antifibrinolytic agents has become more common. These agents have the potential to become embolic and may be associated with an increased risk of vascular occlusion if they enter the systemic circulation, although no study has demonstrated a difference in thrombotic rate with tranexamic acid (TXA) [72,73,74].

#### 3.2.3. Cortical Blindness

Cortical blindness, although rare, is attributable to hypoperfusion of the occipital cortex [2,20,75]. This may occur through ischemic or hemorrhagic events involving the posterior cerebral artery during the procedure.

While ION and CRAO are the most common causes of POVL after spine surgery, POVL has been documented across surgical domains. Recent investigation into the rates of POVL following non-ocular surgery found the incidence per 10,000 to be 8.64 for cardiac surgery, 3.09 for spinal fusion, 1.86 for hip/ femur treatment, 1.24 for colorectal resection, 1.08 for knee replacement, 0.86 for laminectomy without fusion, 0.66 for cholecystectomy, and 0.12 for appendectomy [2]. With respect to cardiac surgery, ION and CRAO remain the most common pathologies; however, the mechanisms often reflect cardiogenic or vascular processes such as aortic insufficiency, transient cerebral ischemia, carotid artery stenosis, embolic stroke, or atrial myxoma, and the risk is elevated with valve surgeries [76,77]. Few cases of POVL following colorectal procedures have been reported and the mechanism remains poorly understood [78,79].

### 3.3. Clinical Presentation

Symptoms generally occur upon awakening from anesthesia but may occur within 48 h of surgery, depending on when the patient becomes alert postoperatively [24,37,49]. Profound, painless central or peripheral vision loss is characteristic of POVL, often occurring bilaterally [56]. Patients frequently present with visual acuity of CF or no light perception (NLP). Reports of NLP were as high as 40.8% in one study, and two additional works described vision of CFs or worse as 70% and 75.8% [32,36,58]. In cases of orbital compression, presentations may involve lid and orbital edema as well as proptosis [14].

## 4. Results

### 4.1. Assessment and Diagnosis

Fundoscopic exam and intracranial MRI are often implicated in the workup of POVL to rule out brain pathology. Orbital MRI is often warranted to assess for optic nerve pathology [56]. Exam findings vary depending on the pathogenesis of the POVL. Patients with ION will exhibit visual field deficits and sluggish pupils [37]. A swollen optic nerve can be visualized on fundoscopic exam for AION, while PION will not have these findings [32,37,80]. The presentation of PION will be consistent with optic neuropathy, but with an unremarkable fundoscopic exam [36]. While PION is often a diagnosis of exclusion, recent reports have argued for the use of diffusion weighted imaging (DWI) to diagnose the acute phase of PION [55,61,65]. DWI has been applied to AION and shows promise in detecting ischemic changes [81]. In the giant cell arteritis population, sensitivity and specificity for DWI in detecting aggregated AION and PION is 87% and 99%, respectively [65]. Fundoscopic exam for CRAO shows a pale and edematous retina coupled with a cherry red macula and the narrowing of arterioles [34,82,83]. Cortical blindness can be identified through a functional pupillary light reflex and unremarkable fundoscopic exam [20,75]. Postoperative MRI can confirm the occurrence of a posterior cerebral artery infarct [62].

### 4.2. Prognosis

Most cases of POVL lead to irreversible damage to the eye, although the prognosis can vary depending on the underlying cause. For patients with ION, prognosis is poor; the severe vision loss associated with PION is often irreparable [35,49,51]. Visual loss was temporary in patients with CRAO, with full recovery in those with retinal infarcts and minor visual disturbances in 50% of those with retinal emboli, albeit following coronary bypass surgery [21]. Cortical blindness may improve, but complete resolution of the visual disturbance is rare [54,63].

### 4.3. Treatment and Prevention

No treatments have demonstrated efficacy in managing POVL [56]. Corticosteroids have been implicated in reducing axonal inflammation, but the patients failed to improve clinically [23,84]. POVL is often irreversible; therefore, prophylactic measures are imperative. A recent meta-analysis of randomized control trials found that IOP was reduced when using propofol based anesthesia, recommending propofol-based total intravenous anesthesia for patients at risk of POVL [59]. A practice advisory in anesthesiology that was updated in 2012 reported that patients can be stratified based on risk [41,51]. High-risk patients were identified as patients undergoing prone spine surgery with preoperative anemia, anticipated long procedures (>6.5 h), or significant blood loss (>44.7% of estimated blood volume) [41,51]. Over 80% of patients undergoing prone spine surgery stated they would like to be informed of the risk of POVL [50]. The advisory suggests that physicians consider warning patients undergoing prolonged procedures or anticipated to have substantial blood loss, or both, that there is an unpredictable risk of POVL [51]. Several case reports indicate that patients with the following conditions are at an elevated risk of POVL: anemia, hypertension, coronary artery disease (CAD), diabetes, smoking history, and obesity [23,26,33,37,52,58,63,64] (Table 2).

With respect to the rarer form of POVL caused by CRAO, the risk factors include hypotension, shock, anemia, longer duration of surgery, and bradycardia [34]. Due to the important role of positioning, some have advocated for the use of three-pin head fixation to eliminate potential orbital pressure [44,53,60,85]. Anatomic variants, such as hypoplasia of a vertebral artery, may increase the risk for cortical blindness, which some authors argue for preoperatively assessing via MR angiography [62]. Nevertheless, there is a paucity of research establishing causal links between POVL and various risk factors [45,51,63]. The strongest evidence for minimizing the risk of POVL involves ensuring no extrinsic orbital compression during positioning, avoiding intraoperative hypotension, minimizing procedural time, and optimizing anesthetic selection to avoid pathological rises in IOP [51,59,64] (Table 3).

## 5. Limitations and Future Directions

Due to the rare nature of POVL, there is a paucity of literature characterizing the complication. The majority of information comes from case reports, which inherently limits the generalizability of the conclusions. This work is a scoping review which offers value in identifying knowledge gaps in the literature but lacks the depth that a systematic review may offer. Furthermore, many of the risk factors described for POVL, such as increased length of procedure and significant blood loss, coincide with challenging, more complicated surgical cases. The stratification of patients by surgical indication, comorbidities, and intraoperative complications may help to eliminate confounding variables when analyzing POVL. Additional work using machine learning algorithms to develop better risk stratification protocols will enable clinicians to offer more informed counseling to patients. Imaging techniques to intraoperatively, or immediately postoperatively, monitor at-risk patients for ischemic ocular events may offer an avenue for detecting POVL prior to it causing devastating, permanent vision changes. While there are currently no treatments for POVL, innovative research focused on the mechanisms comprising POVL can potentially reveal therapeutic targets.

## 6. Conclusions

POVL is a rare and debilitating complication of spine surgery. Patients often show symptoms upon waking from surgery, but there have been reports of symptom onset within 48 h of anesthesia. Patients experience profound vision loss with visual acuity described as CF or HM. Workup for POVL involves a comprehensive fundoscopic exam as well as a brain MRI to assess for intracranial pathology. The pathophysiology underlying POVL is most commonly ION attributable to infarction of the arterial supply to the optic nerve; however, CRAO and cortical blindness also comprise a portion of POVL cases. Due to the ischemic nature of the injury, POVL is frequently associated with irreversible damage to the eye. Risk stratification is vital to identify those most susceptible to POVL to temper expectations given the lack of effective treatments. Patients at the highest risk are those who are undergoing prone spine surgery with either preoperative anemia, a long duration of surgery, and significant blood loss during the case. As visual acuity is correlated with physical activity levels, POVL has significant implications for recovery following surgery. Further research is necessary to establish a robust risk stratification algorithm for POVL and develop effective treatments.

## Figures and Tables

**Table 1:** Historical overview detailing POVL in spine surgery.

Time Period	Main Findings
*1940–1990*	First case reports describing patients with blindness after spine surgery; exam findings are consistent with CRAO. Blindness was also reported following cardiothoracic surgery and general surgery procedures.
*1991–2000*	Additional case reports were published and risk factors for POVL identified: hypotension and prolonged operating time. ION was suspected in conjunction with CRAO to underlie POVL.
*2001–2005*	Prone positioning was identified to increase IOP. Several reviews summarizing case reports documenting ION were published.
*2006–2010*	The American Society of Anesthesiologists Task Force published the first practice advisory on perioperative blindness in 2006. A large national population-based study was published describing visual complications following spine surgery.
*2011–2019*	The 2006 practice advisory was updated in 2012. Comprehensive reviews and case reports on POVL were conducted, with spine surgery noted as one of the most common causes of POVL. The 2012 practice advisory was updated for a second time in 2019.
*2020–present*	Various anesthetics are found to increase IOP, and IOP is noted to be greatest at the end of surgical cases. Additional case reports were published.

**Table 2:** Patient-specific and procedure-specific risk factors for POVL in spine surgery.

Patient-Specific Factors	Procedure-Specific
Anemia [2,58,64]	Prone surgery [2,37,41,51,57,58,64]
Hypertension [2,35,58,63]	Long surgical duration [37,41,51,57,64]
CAD [2,26,35]	Large volume blood loss [30,41,51,57]
Diabetes [2,26,35,63]	Intraoperative hypotension [26,58]
Smoking history [2,26,62]	
Obesity [63,64]	

**Table 3:** Preventative measures to reduce the risk of POVL in spine surgery patients.

Surgical Measures	Anesthesia Measures
Avoid orbital compression when positioning [64]	Management of intraoperative hypotension [41,51,57,64]
Minimize procedural time [41,51,57,64]	Optimize anesthetic selection relative to IOP [59]

## Data Availability

The data presented in this study are available in their respective manuscripts.

## Appendix A

### Search Terms for Blindness or Vision Loss in Spine Surgery

((“spine” [MeSH Terms] OR “spine” [All Fields] OR “spines” [All Fields] OR “spine s” [All Fields] OR (“spinal” [All Fields] OR “spinalization” [All Fields] OR “spinalized” [All Fields] OR “spinally” [All Fields] OR “spinals” [All Fields])) AND (“surgery” [MeSH Subheading] OR “surgery” [All Fields] OR “surgical procedures, operative” [MeSH Terms] OR (“surgical” [All Fields] AND “procedures” [All Fields] AND “operative” [All Fields]) OR “operative surgical procedures” [All Fields] OR “general surgery” [MeSH Terms] OR (“general” [All Fields] AND “surgery” [All Fields]) OR “general surgery” [All Fields] OR “surgery s” [All Fields] OR “surgerys” [All Fields] OR “surgeries” [All Fields] OR “surg*” [All Fields]) AND (“complicances” [All Fields] OR “complicate” [All Fields] OR “complicated” [All Fields] OR “complicates” [All Fields] OR “complicating” [All Fields] OR “complication” [All Fields] OR “complication s” [All Fields] OR “complications” [MeSH Subheading] OR “complications” [All Fields]) AND (“blindness” [MeSH Terms] OR “blindness” [All Fields] OR “blindnesses” [All Fields] OR (“blindness” [MeSH Terms] OR “blindness” [All Fields] OR (“vision” [All Fields] AND “loss” [All Fields]) OR “vision loss” [All Fields]))) AND ((humans[Filter]) AND (english[Filter])).
